# External stimuli-responsive drug delivery to the posterior segment of the eye

**DOI:** 10.1080/10717544.2025.2476140

**Published:** 2025-03-24

**Authors:** Shuting Xu, Yaming Zhang, Jia Li, Xinyu Zhang, Weiping Wang

**Affiliations:** a Department of Pharmacology and Pharmacy, Li Ka Shing Faculty of Medicine, The University of Hong Kong, Hong Kong, China; b State Key Laboratory of Pharmaceutical Biotechnology, The University of Hong Kong, Hong Kong, China; c Laboratory of Molecular Engineering and Nanomedicine, Dr. Li Dak-Sum Research Centre, The University of Hong Kong, Hong Kong, China

**Keywords:** Drug delivery, external stimuli, nanomedicines, photoresponsive nanoparticles, posterior segment eye diseases

## Abstract

Posterior segment eye diseases represent the leading causes of vision impairment and blindness globally. Current therapies still have notable drawbacks, including the need for frequent invasive injections and the associated risks of severe ocular complications. Recently, the utility of external stimuli, such as light, ultrasound, magnetic field, and electric field, has been noted as a promising strategy to enhance drug delivery to the posterior segment of the eye. In this review, we briefly summarize the main physiological barriers against ocular drug delivery, focusing primarily on the recent advancements that utilize external stimuli to improve treatment outcomes for posterior segment eye diseases. The advantages of these external stimuli-responsive drug delivery strategies are discussed, with illustrative examples highlighting improved tissue penetration, enhanced control over drug release, and targeted drug delivery to ocular lesions through minimally invasive routes. Finally, we discuss the challenges and future perspectives in the translational research of external stimuli-responsive drug delivery platforms, aiming to bridge existing gaps toward clinical use.

## 1. Introduction

Posterior segment eye diseases, primarily affecting the vitreous humor, choroid, retina, and retinal pigment epithelium (RPE) of the eye, constitute a significant threat to vision health across all age groups (Cholkar *et al*. 2013, Burton *et al*. 2021). These disorders include, but are not limited to, inherited retinal degeneration, neovascular eye diseases, inflammatory and neurodegenerative eye diseases, and intraocular tumors (Wu *et al*. 2024, Nayak and Misra 2018, Gabai *et al*. 2023). Notably, over the past decade, the aging global population has led to an increasing prevalence of age-related and chronic eye disorders, including age-related macular degeneration (AMD), which is currently recognized as the third leading cause of irreversible vision impairment among the elderly (Wong *et al*. 2014, Sarkar and Dyawanapelly 2021, Mitchell *et al*. 2018). It is projected that around 500 million people will be affected by AMD and diabetic retinopathy (DR), two major fundus neovascularization diseases in the elderly, in the coming two decades (Wong *et al*. 2014, Zhou *et al*. 2024, Saeedi *et al*. 2019). Deteriorating vision can severely affect various aspects of patients’ daily lives, including self-care, employment, and mental health (Langelaan *et al*. 2007). Furthermore, the rising prevalence of blinding disorders has imposed a growing burden on healthcare systems worldwide (Swenor and Ehrlich 2021). Consequently, developing effective therapeutic strategies to manage these diseases is urgently essential.

Unlike ocular surface diseases, where topical formulations, such as eyedrops, effectively reach lesions, delivering drugs to the back of the eye presents a tough challenge (Geroski and Edelhauser 2000). The intricate physiological barriers of the eye significantly limit drug penetration through ocular tissues (Li *et al*. 2023). Furthermore, dynamic elimination mechanisms result in the short half-lives of administrated drugs, necessitating frequent dosing (Ahmed *et al*. 2023, Grassiri *et al*. 2021). Most existing therapeutics for posterior segment eye disorders rely on routine intravitreal administration. For example, intravitreal injections of anti-vascular endothelial growth factor (anti-VEGF) agents are the first-line therapy for exudative AMD, and dexamethasone implants are utilized to treat macular edema and noninfectious uveitis (Ghanchi *et al*. 2022, Del Amo and Urtti 2008, Scaramuzzi *et al*. 2015). However, this invasive administration method diminishes patient compliance and carries the risk of adverse ocular complications, such as ocular hypertension, endophthalmitis, and vitreous hemorrhage (van der Reis *et al*. 2011, Falavarjani and Nguyen 2013). The need for frequent dosing in chronic conditions also leads to an increased likelihood of adverse events (Falavarjani and Nguyen 2013). Therefore, there have been unmet clinical needs for developing drug delivery platforms with improved efficiency and minimized invasiveness.

To date, growing research has increasingly focused on utilizing stimuli to overcome biological barriers and improve the pharmacokinetic profiles of drugs, representing a promising approach to enhance drug delivery to the posterior side (Lyu *et al*. 2021, Lin *et al*. 2021, Wang *et al*. 2023, Huang *et al*. 2018). For instance, certain physiological or pathological conditions can be used as a cue to trigger the localized retention or drug release from nanocarriers, including redox gradients, pH, ionic strength, temperature, enzyme levels, etc (Lin *et al*. 2021, Fleige *et al*. 2012, Mura *et al*. 2013). Such nanocarriers with predictable and preprogrammed reactions to specific endogenous signals are commonly referred to as endogenous stimuli-responsive systems (Fleige *et al*. 2012). Several recent reviews have elaborated their applications in eye disease therapies (Lyu *et al*. 2021, Lin *et al*. 2021, Wang *et al*. 2023,  Lynch *et al*. 2020). Alternatively, drug delivery can be remotely controlled by exogenous stimuli. Commonly employed external stimuli, such as light, ultrasound, magnetic field, and electric current, offer advantageous features, including minimal invasiveness, precise spatiotemporal control, and high adjustability (Wang *et al*. 2023, Liao *et al*. 2024). Drug release from traditional nanocarriers (e.g., liposomes and polymeric nanoparticles) typically occurs through direct diffusion or carrier degradation (Mitchell *et al*. 2021, Imperiale *et al*. 2018). These methods often lack precise dosage control and may not meet the demands of personalized treatment (Mitchell *et al*. 2021). Developing drug delivery platforms that leverage stimuli-responsive materials enables targeted delivery and controllable release of payloads in response to external triggers, which is the focus of the topic of this review (Lyu *et al*. 2021, Karimi *et al*. 2016, Wang *et al*. 2023).

Among various external stimuli, light-responsive drug delivery strategies have been more extensively studied, owing to the light-receptive nature of the eye and the availability of diverse photoresponsive nanomaterials (Lin *et al*. 2021, Singh *et al*. 2024). Additionally, integrating photoactive agents into nanosystems facilitates multifunctional therapies, such as combining drug delivery with photodynamic therapy and photothermal therapy (Yang *et al*. 2024). Other external stimuli, such as magnetic or electric fields, can generate forces on magnetic materials or ionic species, respectively, which have been utilized to guide drug transport across the sclera and vitreous humor (Huang *et al*. 2018). Similarly, ultrasound has been employed to temporarily disrupt ocular barriers, thereby enhancing targeted delivery to the posterior segment (Huang *et al*. 2018, Rousou *et al*. 2021). These applications hold significant promise for improving intraocular drug bioavailability and the specificity of drug action, and enabling precise dosage adjustments (Wang *et al*. 2023, Li *et al*. 2020).

**Figure 1.**
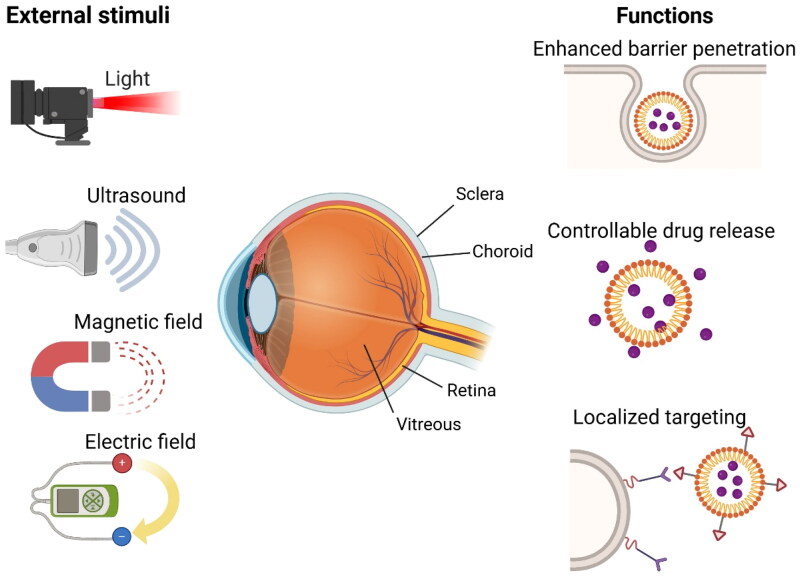
Schematic illustration of external stimuli-responsive drug delivery strategies for the treatment of posterior segment eye diseases. Commonly used external stimuli, such as light, ultrasound, magnetic field, and electric field, have demonstrated significant potential in enhancing the penetration of administered drugs across biological barriers, enabling precise control over drug release or localized targeting of nanocarriers. The image was created with BioRender.com.

This review provides an overview of common drug administration routes and physiological barriers associated with ocular drug delivery, and specifically focuses on emerging advances in external stimuli-responsive drug delivery strategies for the treatment of posterior segment eye diseases (Figure 1). We examine the fundamental design principles of these intelligent drug delivery platforms and illustrate how they effectively address the unique challenges in ocular drug delivery through representative examples. In the end, we discuss future research directions and identify the key obstacles that need to be overcome for the clinical translation of these novel technologies.

## 2. Common administration routes and physiological barriers for drug delivery to the posterior segment of the eye

**Figure 2.**
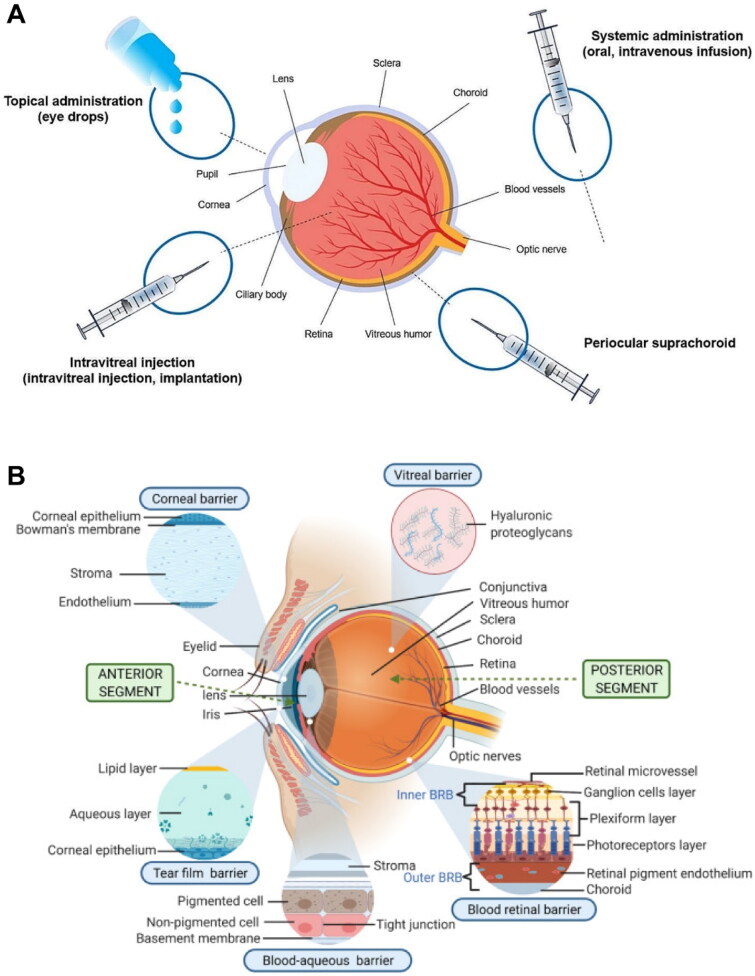
(A) Schematic illustration of common administration routes for ocular drug delivery. Reproduced from Liu *et al*. (2024) with permission. Copyright: 2024 the authors. Journal of Nanobiotechnology published by Springer Nature. (B) Schematic representation of physiological barriers of the eye. Reproduced from Adrianto *et al*. (2022) with permission. Copyright: 2021 the authors. Drug Delivery and Translational Research published by Springer Nature.

Ocular drug administration mainly includes topical, periocular, intravitreal, and systemic routes (Figure 2A) (Bisht *et al*. 2018). While topical formulations are preferable for treating eye diseases, conventional topical medications have difficulty reaching the fundus, typically achieving less than 5% of intraocular bioavailability (Gabai *et al*. 2023, Cabrera *et al*. 2019). Intravitreal administration and periocular methods, such as sub-Tenon, subconjunctival, retrobulbar, suprachoroidal, and posterior-juxtascleral injections, have become the primary approaches for managing posterior segment eye diseases (Del Amo *et al*. 2017, Rowe-Rendleman *et al*. 2014, Agban *et al*. 2019). These methods circumvent anatomical barriers like the cornea and conjunctiva, resulting in initially high drug concentrations in the posterior compartments, such as the choroid and retina (Tavakoli *et al*. 2020). Common systemic routes involve intravenous injection and oral route. However, they are rarely used for treating eye diseases due to the extremely low percentage of intravenous drugs that could reach the vitreous (< 2%) (Gaudana *et al*. 2010).

The anatomical structures and physiological barriers of the eye have been thoroughly reviewed in several recent reviews (Figure 2B) (Lyu *et al*. 2021, Huang *et al*. 2018, Liu *et al*. 2024). Collectively, the tear film, cornea, and blood-aqueous barriers (BABs) act as primary defensive barriers against topical drug delivery (Qi *et al*. 2023). The minimal resident tear volume (approximately 7 μL), blinking reflex, rapid tear turnover (around 10 μL/min), and naso-lacrimal drainage result in significant drainage of eyedrops, with more than 95% being lost (Weng *et al*. 2017, Subrizi *et al*. 2019). Additionally, the lipophilic and tightly jointed corneal epithelium typically hinders the transport of hydrophilic drugs, while the hydrophilic corneal stroma resists the penetration of hydrophobic drugs (Afarid *et al*. 2022, Liu *et al*. 2024). The BABs consist of highly organized iris vascular endothelium and ciliary body epithelium (Li *et al*. 2023, Han *et al*. 2023). Together with vitreous and aqueous humor turnover, they function as dynamic barriers that facilitate rapid anterior elimination of drugs (Li *et al*. 2023, Han *et al*. 2023).

The vitreous body, lying in the posterior segment of the eyeball, is mainly composed of water, collagen fibers, and proteoglycans (Lyu *et al*. 2021). Since the pore size of the vitreous cavity is around 500 nm, microparticles are more likely to be restricted in the vitreous cavity (Lyu *et al*. 2021, Moisseiev and Loewenstein 2017). Furthermore, the diffusion of positively charged nanoparticles is hindered by electrostatic interactions with the negatively charged vitreous matrix (Han *et al*. 2023). The inner limiting membrane, characterized by the negative charge nature and limited pore size of around 10 nm, acts as the innermost static barrier for intravitreal drug transport to the retina (Huang *et al*. 2018, Del Amo *et al*. 2017). Likewise, the retinal bioavailability of intravitreally injected agents is also subject to anterior elimination pathways, such as aqueous humor outflow and lymphatic clearance (Del Amo *et al*. 2017). Furthermore, the choroid, located between the retinal pigment epithelium (RPE) and the sclera, has a rich blood supply, which leads to significant drug elimination by systemic absorption (Hughes *et al*. 2005).

Despite the rich blood flow of choroidal vasculature, systemic drug delivery to the posterior side of the eye is arduous due to the presence of the blood-retina barriers (BRBs) (Vellonen *et al*. 2018). The BRBs consist not only of densely packed retinal capillary endothelium (inner barriers) and RPE (outer barriers) but alsoof diverse active efflux transporters, such as P-glycoproteins and multidrug resistance proteins (Liu *et al*. 2024, Agban *et al*. 2019). Noteworthily, metabolism and excretion by the liver and kidney mainly contribute to the elimination of intravenously administrated agents (Zhang *et al*. 2021). Hence, enhancing the ocular targeting efficiency while minimizing systemic side effects has become the primary goal for systemic drug delivery.

## 3. External stimuli-responsive drug delivery to the posterior segment of the eye

In this section, we review recent advancements and application prospects of using external stimuli for treating posterior segment eye diseases, including light, ultrasound, magnetic field, and electric field.

### 3.1. Light-responsive drug delivery

Given that the intrinsic anatomical structures of eyeballs allow for efficient light transmittance, light irradiation is an attractive external stimulus for controlling drug-release processes in the eye (Pearson *et al*. 2021). Light irradiation is noninvasive and enables spatiotemporal and multiparameter controllability (Pearson *et al*. 2021). Its therapeutic applications are convenient and cost-efficient, owing to the development of portable and wearable light sources (Lee *et al*. 2020). Light is generally classified based on its wavelength: ultraviolet (UV) light (200 to 400 nm), visible light (400 to 700 nm), and near-infrared (NIR) light (700 to 1000 nm) (Lin *et al*. 2021). Short-wavelength light, such as UV and blue light, possesses higher energy but is limited by poor tissue penetration and potential tissue damage (Rapp and DeForest 2021, Abdelmohsen *et al*. 2023). Additionally, light with wavelengths shorter than 650 nm is significantly affected by hemoglobin absorption, while NIR light with wavelengths beyond 900 nm is primarily undermined by water absorption (Rwei *et al*. 2015). Consequently, red and NIR light with wavelengths between 650 and 900 nm is preferred for biomedical applications due to its superior tissue penetration, lower absorption rates, and minimal risk of causing tissue damage (Abdelmohsen *et al*. 2023, Lane *et al*. 2018).

In recent decades, various light-responsive or photoresponsive drug delivery systems have been developed to treat posterior segment eye diseases (Wu *et al*. 2024, Lin *et al*. 2021, Wang *et al*. 2023). These systems function through different photochemical mechanisms, including photoisomerization, photodimerization, photoaddition, and photocleavage (Fomina *et al*. 2012). Representative studies are listed in Table 1. In addition to triggering targeting effect or initiating drug release, light irradiation can induce therapeutic effects of photoactive agents, including exerting photodynamic and photothermal effects, which offers additional benefits for disease treatments (Abdelmohsen *et al*. 2023, Xu *et al*. 2023).

**Table 1.** Summary of representative examples of light-responsive ocular drug delivery utilizing photochemical mechanisms.

Photoresponsive moiety	Light irradiation parameter	Mechanism controlling drug delivery	Animal model	Reference
Azobenzene	UV (275–400 nm), 10 min using 400 W high-pressure Hg lamp	Triggered the release of rhodamine B as the model drug	Rats	(Geng *et al*. 2017)
Azobenzene	480 nm, 1 × 10^16^ photons/cm^2^ /s, 15 s	Controlled light sensitivity of retinal ganglion cells with a photoresponsive P2X receptor agonist	Retinal degeneration 1 (rd1) mice	(Tochitsky *et al*. 2016)
Azobenzene	450 nm, 1 mW/cm^2^	Controlled light sensitivity of retinal ganglion cells with a photoresponsive agonist of voltage-gated ion channels	rd1 mice	(Cao *et al*. 2021)
Nitrobenzyl ester	365 nm, 8 mW/cm^2^, 5 min	Triggered the release of an anti-angiogenic agent from an intravitreal-injected depot	Laser-induced choroidal neovascularization (CNV) rats	(Huu *et al*. 2015)
Nitrobenzyl ester	400–430 nm, 0.2 mW/cm^2^, 10 min	Triggered the release of a β-adrenergic receptor inhibitor from contact lenses	Glaucoma model mice	(Mu *et al*. 2018)
Coumarin	254 nm, 4.5 mW/cm^2^, 5 min	Triggered the release of 5-fluorouracil from copolymer-coated intraocular lens surface	Posterior capsular opacification rabbit model	(Xia *et al*. 2022)
Coumarin	400 nm, 50 mW/cm^2^, 3 min	Triggered the localized targeting effect of nanocarriers	Laser-induced CNV mice	(Liu *et al*. 2022)
Coumarin	505 nm, 50 mW/cm^2^, 5 min	Triggered the release of the chemotherapeutic drug from nanocarriers	Retinoblastoma-bearing mice	(Long *et al*. 2021)
Cyanine	690 nm, 80 mW/cm^2^, 5 min	Triggered the activation of photoresponsive prodrug nanoparticles	Laser-induced CNV mice	(Xu *et al*. 2024)

#### 3.1.1. Light-responsive drug delivery based on photoisomerization mechanism

Photoisomerization is a reversible process involving cis/trans or ring-closed/opening conformational changes in alkene or spirocyclic structures upon exposure to ultraviolet or visible (UV-Vis) light (Kondo 2020, Vlasceanu *et al*. 2018). Representative photoisomerization groups include azobenzene (AZO), stilbene, and spiropyran (Marturano *et al*. 2016). In a study by Geng *et al*., photoresponsive vesicles were fabricated using a lipid-AZO derivative and sodium dodecyl sulfate (SDS) to control the release of rhodamine B, a model drug, in rat eyes upon UV light irradiation (275–400 nm) (Geng *et al*. 2017). Light-sensitive liposomes and micelles have also been designed by integrating photoisomerizable moieties into the lipid or copolymer structures (Barhoumi *et al*. 2015, Morgan *et al*. 1987, Ohya *et al*. 1998, Han *et al*. 2013). For instance, Bisby *et al*. fabricated a photosensitive liposome system containing 25% (molar ratio) of AZO-incorporating acyl chains (Bisby *et al*. 2000). These liposomes displayed significantly increased payload release upon exposure to 355 nm UV light irradiation (15 mJ) for nanoseconds, or 470 nm blue light irradiation (20 mW/cm^2^) within one minute (Bisby *et al*. 2000). However, there is still a lack of testing for such photoisomerization-based depot systems for controlled drug release in ophthalmic disease models.

#### 3.1.2. Light-responsive drug delivery based on photodimerization and photoaddition mechanism

Anthracene and cinnamic acid, the most commonly used photodimerization moieties, undergo reversible intermolecular cycloadditions under UV-Vis light irradiation (Kondo 2020). Wells *et al.* demonstrated that 365 nm UV irradiation caused increased cargo release from pegylated anthracene-grafted hyaluronan hydrogels *in vitro* as a result of the photodimerization of anthracene (Wells *et al*. 2011). To improve the visible light responsiveness of anthracene-based ­hydrogel, Jiang *et al*. designed benzyl imine-substituted anthracene for preparing supramolecular hydrogel, which demonstrated a significant red-shift in absorption to 400–470 nm and exhibited fast photochemical kinetics *in vitro* (Jiang *et al*. 2020).

On the other hand, UV irradiation-induced photoaddition reactions include [4 + 4]-cycloaddition of anthracenes, [2 + 2]-cycloaddition of cinnamates, stilbene and maleimides, as well as thiol-ene click reactions (Li *et al*. 2019). These mechanisms have been explored to fabricate light-activatable *in situ* gelling systems. Recently, Hu *et al*. utilized the [2 + 2]-cycloaddition reaction between a coumarin derivative and 5-fluorouracil (5-FU) to design a photo-controllable implant for posterior capsular opacification therapy. Light irradiation at 365 nm for 2 h successfully grafted 5-FU to the polymer material. Subsequently, 254 nm light irradiation induced the on-demand release of 5-FU (4.5 mW/cm^2^ for 5 min) after implantation in rabbit eyes (Hu *et al*. 2024).

#### 3.1.3. Light-responsive drug delivery based on photocleavage mechanism

**Figure 3.**
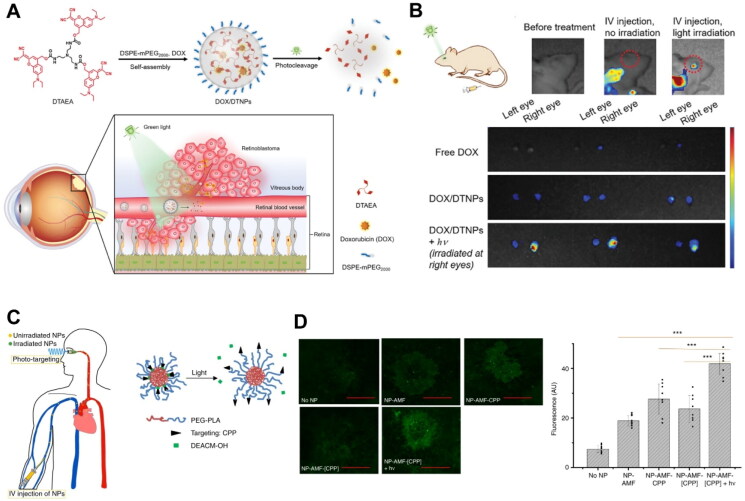
Representative examples of light-responsive drug delivery based on the photocleavage mechanisms for the treatment of posterior segment eye diseases. (A) Schematic representation of light-controlled intraocular drug release from a photoresponsive nanosystem for retinoblastoma therapy. (B) Schematic illustration and representative images showing *in vivo* biodistribution of the photoresponsive nanoparticles 1 h after intravenous administration, with or without light irradiation to the mouse eyes. (A-B) Reproduced from Long et al. (2021) with permission. Copyright: 2021 the authors. Advanced Science published by Wiley-VCH GmbH. (C) Schematic representation of light-triggered targeting of intravenously injected nanoparticles to the eye. (D) Representative confocal images and quantitative analysis of choroid-RPE flat-mounts showing light-triggered accumulation of fluorescein (AMF)-loaded nanoparticles (NP-AMF-[CPP]) in CNV lesions. Data were presented as mean ± standard deviation (*n* = 8). (C-D) Reproduced from Wang et al. (2019) with permission. Copyright: 2019 the authors. Nature Communications published by Springer Nature.

Most photo-mediated drug release strategies integrate photocleavable moieties (e.g., nitrobenzyl ester, coumarin, cyanine, and boron-dipyrromethene (BODIPY)) into nanosystem design (Rwei *et al*. 2015, Liu *et al*. 2022). The design principles include photocleavable moieties as the backbone or conjugation linkers of nanocarriers, along with photocleavable prodrugs encapsulated within these nanocarriers (Liu *et al*. 2022). For instance, Huu *et al*. developed a UV light-degradable polymeric nanoparticle depot composed of nitrobenzyl moiety-containing monomer. The nano-depot underwent self-immolation under 365 nm UV irradiation, leading to its decomposition and the release of the anti-angiogenic agent nintedanib in choroidal neovascularization (CNV) mouse eyes (Huu *et al*. 2015). Qi *et al*. designed a photo-labile micelle system with pendant hydroxyl groups protected by [7-(diethylamino)coumarin-4-yl]methyl (DEACM). Blue light irradiation (400 nm, 50 mW/cm^2^, 3 min) triggered the swelling of micelles and accelerated the payload release (Qi *et al*. 2024). A green light-responsive self-assembled nanosystem was further developed for retinoblastoma treatment (Figure 3A and B). The nanosystem was based on a photocleavable clathrin-like trigonal molecule. Photolysis of the trigonal molecule under 505 nm light irradiation (50 mW/cm^2^, 5 min) destabilized the nanosystem. This led to the release of encapsulated doxorubicin and enabled efficient drug penetration through the retina blood vessels (Long *et al*. 2021).

Moreover, photocleavable moieties have been used to mask the targeting ligands of nanosystems. Such nanosystems are typically referred to as ‘photo-targeted systems’ (Liu *et al*. 2022). Using this approach, the nanosystems can be designed to penetrate specific tissues exposed to light irradiation, thereby minimizing unwanted off-target effects (Dvir *et al*. 2010). For example, Wang *et al*. proposed intravenous photo-targeted polymeric nanoparticles (NP-[CPP]) for light-triggered targeting accumulation in the back of the eye (Figure 3C and D). The nanoparticles were modified with DEACM-caged cell-penetrating peptide (CPP). Green light (400 nm) irradiation to CNV mouse eyes could trigger the removal of the protecting group, enhancing the accumulation of activated nanoparticles in the diseased choroid (Wang *et al*. 2019).

#### 3.1.4. Light-responsive drug delivery based on photothermal and photodynamic effects

Beyond photochemical reactions, facile designs utilize heat and reactive oxygen species (ROS) generated by photothermal agents and photosensitizers, respectively, for controlled drug release (Zhou *et al*. 2018, Zhao *et al*. 2019, Zhao *et al*. 2019).

**Figure 4.**
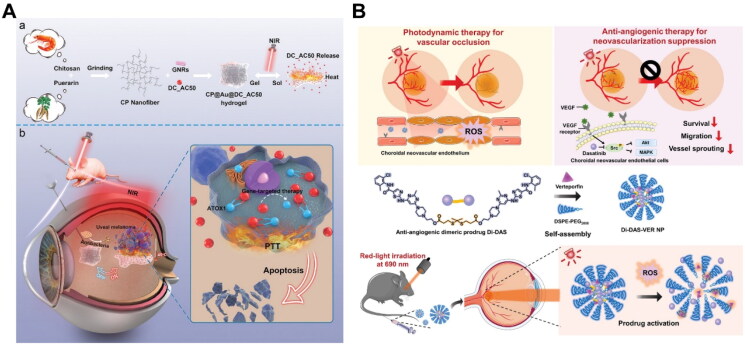
Representative examples of light-responsive drug delivery based on photothermal and photodynamic effects for the treatment of posterior segment eye diseases. (A) Schematic illustration of a photoresponsive hydrogel system containing gold nanorods as the photothermal agent and an ATOX1 inhibitor for the combination treatment of uveal melanoma. Reproduced from Wang *et al*. (2021) with permission. Copyright: 2021 the authors. Advanced Science published by Wiley-VCH GmbH. (B) Schematic representation of a photoresponsive prodrug nanosystem composed of a ROS-sensitive anti-angiogenic prodrug and a photosensitizer for the combination treatment of AMD. Reproduced from Xu *et al*. (2023) with permission. Copyright: 2023 the authors. Advanced Science published by Wiley-VCH GmbH.

To take advantage of the photothermal effect to trigger drug release, Basuki *et al*. constructed a photoresponsive agarose hydrogel system containing polymer-functionalized gold nanoparticles (AuNPs) and anti-VEGF antibody bevacizumab (Basuki *et al*. 2017). After implanting the AuNPs/hydrogel depots into the anterior chamber of bovine eyes, visible light illumination (0.5 W, 10 min) could activate the photothermal effects of loaded AuNPs. The subsequent temperature increase led to reversible softening of agarose hydrogel matrix after reaching its melting point (45 °C), releasing the pre-loaded FITC-BSA protein (Basuki *et al*. 2017). Significantly higher fluorescence intensity of FITC-BSA was detected in the bovine aqueous humor after light irradiation, which indicated the pre-loaded protein passively diffused inside the eye (Basuki *et al*. 2017). Such light-responsive hydrogel depot could be applied for the repetitive release of anti-VEGF proteins to treat AMD and diabetic retinopathy. Similarly, Wang and colleagues constructed a multifunctional antibacterial hydrogel system consisting of gold nanorods and ATOX1 inhibitor DC_AC50 for uveal melanoma treatment (Figure 4A) (Wang *et al*. 2021). The novel hydrogel system enabled photothermal effect and on-demand release of DC_AC50 simultaneously upon NIR light illumination (808 nm, 0.5 W/cm^2^, 5 min) (Wang *et al*. 2021). Another study utilized zinc oxide-modified biochar (ZnO-BC) as the photothermal agent and integrated it with sodium alginate-chitosan hydrogels for photo-controlled drug release to treat glaucoma (Wang *et al*. 2021). Chen *et al.* designed a NIR-light-responsive drug-eluting intraocular lens containing thermosensitive polymer brushes, photothermal agent indocyanine green (ICG), and chemotherapeutic agent doxorubicin. Light irradiation at 808 nm (0.38 W/cm^2^, 5 min) could effectively trigger the photothermal effect and drug release in rabbit eyes against posterior capsular opacification (Qin *et al*. 2023).

Although metallic materials like gold nanoparticles have been utilized for photothermally induced drug release in the eye, biocompatibility issues must be considered in the design of these nanosystems. Research has shown that the toxicity of gold nanoparticles is associated with their physiochemical properties, including particle size, shape, surface charge and ­functionalization (Guerrero *et al*. 2014, Zhu *et al*. 2019). For example, cationic gold nanoparticles with an ultrasmall size (1.3 nm) demonstrated concentration-dependent toxicity on zebrafish retinal pigmentation (Zhu *et al*. 2019, Kim *et al*. 2013). While no signs of optic nerve toxicity were observed during one-month monitoring after intravitreal injection of a high dose of gold nanoparticles (670 μmol, 0.1 mL) in rabbit eyes (Bakri *et al*. 2008), long-term toxicity studies are still needed to ensure the safety of using gold nanoparticles as therapeutics.

The mechanism of photodynamic therapy is based on ROS generation by photosensitizers, which are already used for the selective ablation of neovascular lesions and tumors (Renno and Miller 2001). Xu *et al.* developed light-responsive self-assembled nanoparticles composed of a ROS-sensitive anti-angiogenic prodrug and verteporfin for intravenous treatment of CNV (Figure 4B) (Xu *et al*. 2023). The light-triggered photodynamic effect simultaneously led to the cleavage of ROS-sensitive prodrug and triggered intraocular drug accumulation (Xu *et al*. 2023). The strategy of leveraging photodynamic and photothermal effects achieves controlled drug release and extra therapeutic benefits. This approach represents a promising alternative for treating fundus neovascularization diseases and ocular tumors, with potential enhancement through the combination of phototherapies.

### 3.2. Ultrasound-responsive drug delivery

Ultrasound refers to vibrating sound waves with frequencies over 20 kHz, which are beyond the audible range for humans (Schroeder *et al*. 2009). Ultrasound is highly regarded for its biosafety, non-ionizing nature, deep tissue penetration, and high spatial resolution (Entzian and Aigner 2021). It has long been employed in clinics for imaging and lithotripsy (Rousou *et al*. 2021). Based on the frequency spectrum, ultrasound can be categorized into two distinct types: low-frequency (20 to 100 kHz) and high-frequency (over 1 MHz) (Lafond *et al*. 2017). Generally, low-frequency ultrasound exhibits deeper penetration depth but has an unfocused working area. In contrast, high-frequency ultrasound can be focused on a small area. However, high-frequency ultrasound produces a weaker cavitation effect, making it less effective in disrupting drug carriers or biological barriers (Rousou *et al*. 2021, Miller *et al*. 2012, Xia *et al*. 2016).

Most therapeutic applications of ultrasound currently focus on enhancing drug delivery to the posterior segment by temporarily increasing the permeability of biological barriers (Rousou *et al*. 2021). The underlying mechanism involves the generation of oscillating/collapsing microbubbles that pose mechanical forces on the neighboring cell membranes (Rousou *et al*. 2021, Deprez *et al*. 2021). This process leads to the formation of pores (termed as sonoporation), which perturbs intercellular junctions or induce endocytosis, ultimately increasing penetration of administrated agents through biological barriers (Rousou *et al*. 2021, Deprez *et al*. 2021). In general, low-intensity ultrasound results in stable cavitation, whereas focused or high-intensity focused ultrasound is more likely to generate inertial cavitation and thermal effect (Sirsi and Borden 2014, Ter Haar 2007).

The combined application of ultrasound and microbubbles has been shown to enhance transscleral drug delivery or intravitreal drug transport to the retina and choroid (Lin *et al*. 2021, Huang *et al*. 2018, Suen *et al*. 2013, Cheung *et al*. 2010). Representative studies and experimental conditions are listed in Table 2. For example, Chau *et al*. demonstrated that ultrasound at 40 kHz (0.05 W/cm^2^, 30 s) significantly improved the scleral penetration of dextran (20 to 150 kDa) in *ex vivo* rabbit eyes compared to other tested parameters, including 500 kHz, 1 MHz, and 3 MHz (Chau *et al*. 2017). Stable cavitation induced by low-intensity ultrasound is the main mechanism contributing to significant intrascleral accumulation of dextran (Chau *et al*. 2017). Zheng *et al*. found that ultrasound-mediated microbubble destruction (1 MHz, 2 W/cm^2^, 300 s) enhanced adenoviral transfection of intravitreal TGF-β2 siRNA/PDGF-B siRNA rAAV2 vectors in the rat retina (Zheng *et al*. 2012). Recently, Huang *et al*. reported that transscleral ultrasound application (1 MHz, 0.5 W/cm^2^, 30 s) enhanced the vitreous mobility and retinal accumulation of Connexin43 mimetic peptide (Cx43)-loaded human serum albumin (HSA) nanoparticles following intravitreal injection, without causing detectable retinal damage in an *ex viv*o bovine eye model. More than 50% of HSA nanoparticles remained on the retina 7 days after intravitreal injection and ultrasound application (Huang *et al*. 2017).

**Table 2.** Summary of representative examples of ultrasound-responsive ocular drug delivery.

Ultrasound parameter	Aim of using ultrasound	Animal model	Reference
400 kHz or 600 kHz, 0.8 W/cm^2^, 5 min	Increased drug concentrations in aqueous humor after topical application of dexamethasone sodium phosphate	Rabbits	(Nabili *et al*. 2014)
1 MHz, 50% duty cycle, power of 1, 1.5, or 2 W/cm^2^, 15 to 120 s, perflutren as the contrast agent	Increased gene transfer of intraocularly injected plasmid to the retina	Rabbits	(Sonoda *et al*. 2006)
1 MHz, 50% duty cycle, power of 2 W/cm^2^, 5 min in total, SonoVue^®^ as the contrast agent	Increased gene transfer of subretinally injected Adeno-associated virus (AAV) vector to the retina	Rats	(Li *et al*. 2009)
0.3 MHz, power of 0.5, 1.0. 2.0 or 2.5 W/cm^2^, 60 s, perfluoropropane as the contrast agent	Increased gene transfer of intravitreally injected AAV to the retina	Rats	(Xie *et al*. 2010)
3 MHz, 6% duty cycle, power of 0.15 W/cm^2^, 60 s, perfluoropropane as the contrast agent	Increased gene transfer of intravitreally injected plasmid to the retina	Rabbits	(Sonoda *et al*. 2012)
1 MHz, 0 to 3.0 W/cm^2^, 60 s, SonoVue^®^ as the contrast agent	Improved the transport of intravitreally injected nerve growth factor to the retina	Rabbits	(Shen *et al*. 2016)
40 kHz, 500 kHz, 1 MHz or 3 MHz, 0.05 W/cm^2^, 30 s	Increased transscleral transport of macromolecules	*Ex vivo* rabbit eyes	(Chau *et al*. 2017)
1 or 3 MHz, 0.05 W/cm^2^, 30 s	Increased transscleral transport of proteins	*Ex vivo* rabbit eyes	(Cheung *et al*. 2010)
1 MHz, 0.5 W/cm^2^, 30 s	Increased the nanoparticle penetration across the retina after intravitreal injection	*Ex vivo* bovine eyes	(Huang *et al*. 2017)
1 MHz, 0 to 2.5 W/cm^2^, 50 to 100% duty, 60 s, perfluoropropane as the contrast agent	Improved the migration of intravitreally injected nanobubbles to the posterior side of the vitreous	*Ex vivo* bovine and porcine eyes	(Thakur *et al*. 2019)
0.3 to 0.6 MPa, 120 s (10 ms burst), focused ultrasound with microbubbles	Increased the BRB penetration of intravenously injected nanoparticles	Mice	(Park *et al*. 2024)
1 MHz, 2.37 W/cm^2^, 30% duty cycle, 5 min each time for 3 times	Increased the transscleral and transconjunctival transport of fluorescein sodium to the posterior segment	Rabbits	(You *et al*. 2023)
400 kHz or 3 MHz, 1 W/cm^2^, 5 min	Increased transscleral delivery of anti-VEGF proteins	*Ex vivo* rabbit eyes	(Almogbil *et al*. 2021)

Moreover, ultrasound-responsive delivery systems provide an interesting approach for enhancing intravenous drug delivery to the retina by reversible and selective modulation of the BRB opening (Park *et al*. 2012, Bourdin *et al*. 2023). Cavitating microbubbles convert acoustic energy into mechanical pressure and stress on vessel walls, leading to the perturbation of intercellular junctions and enhanced BRB penetration (Rousou *et al*. 2021, Rad *et al*. 2023). Studies on stable cavitation-mediated BRB disruption have proven safety and reversibility, and ultrasound-induced paracellular diffusion of hydrophobic small molecules could last for several hours (Rousou *et al*. 2021, Khalil *et al*. 2023). Recent reports have shown that focused ultrasound to the retina can enhance the transport of intravenously injected macromolecules (e.g., Evans blue, IgG, IgM) and gold nanoparticles across the BRB, facilitating their delivery to the inner nuclear and ganglion cell layers (Park *et al*. 2024, Touahri *et al*. 2020). Park *et al*. studied the impact of pressure amplitudes of ultrasound application (0.69 MHz, 60 s) on the BRB permeation in rats. Despite the reversibility of BRB opening mediated by 0.88 MPa ultrasound, a higher amplitude (1.1 MPa) of focused ultrasound caused notable retinal damage, including erythrocyte extravasation (Park *et al*. 2012).

Moreover, ultrasound has been noted to promote the drug release from drug delivery systems, including microbubbles, liposomes, nanodrops, micelles and microemulsions (Chandan *et al*. 2020). The mechanisms underlying ultrasound-triggered drug release are complex, involving cavitation effects, mechanical force-induced disruption of nanocarriers, and ultrasound-induced thermal effects (Sirsi and Borden 2014, Zhang *et al*. 2016, An *et al*. 2023). To improve sonication responsiveness, phase-change contrast agents such as perfluorocarbons (PFCs) have been employed to physically compromise the integrity of nanocarriers by generating shear forces through the vaporization of these agents (Sirsi and Borden 2014, Silverman *et al*. 2022). Besides, sonosensitizers have been used to induce drug release through ROS production, which disrupts the chemical bonds of carriers (An *et al*. 2023). Although ultrasound-triggered drug release strategy holds promise for enhancing drug accumulation in diseased lesions, evidence supporting its use in ophthalmic disease models remains limited.

The utilization of the ultrasound-assisted drug delivery strategy also raises some biosafety concerns due to potential hazards associated with microbubble cavitation, including severe disruption of the endothelial cytoskeleton, hyperthermia, and other adverse events (Touahri *et al*. 2020, He *et al*. 2022). Ultrasound frequencies below 400 kHz are considered safe for the human eyes due to their minimal thermal effects (Yang *et al*. 2022). Comprehensive evaluations are necessary to assess the impact of low-frequency and high-intensity ultrasound on ocular tissues in preclinical research.

### 3.3. Magnetic field-responsive drug delivery

Magnetic fields with frequencies less than 300 Hz exhibit little tissue absorption, and have been widely used for biomedical applications, such as diagnostic imaging and targeted drug delivery (Armenia *et al*. 2022). Magnetic fields are an effective tool for remotely guiding and dynamically controlling the penetration of nanocarriers through biological barriers via magneto-mechanical forces (Lyu *et al*. 2021, Wang *et al*. 2023). The design of magnetic field-responsive drug delivery systems typically incorporates magnetic metals, such as iron, manganese, cobalt, and nickel. Additionally, magnetic materials exhibit a prominent magnetocaloric effect under alternating magnetic fields due to Néel relaxation and hysteresis loss (Shi *et al*. 2015). Therefore, combining magnetic agents with temperature-sensitive materials enables controlled drug release through thermo-triggered phase changes in the temperature-sensitive matrix under an alternating magnetic field (Armenia *et al*. 2022, Chiu-Lam and Rinaldi 2016).

**Figure 5.**
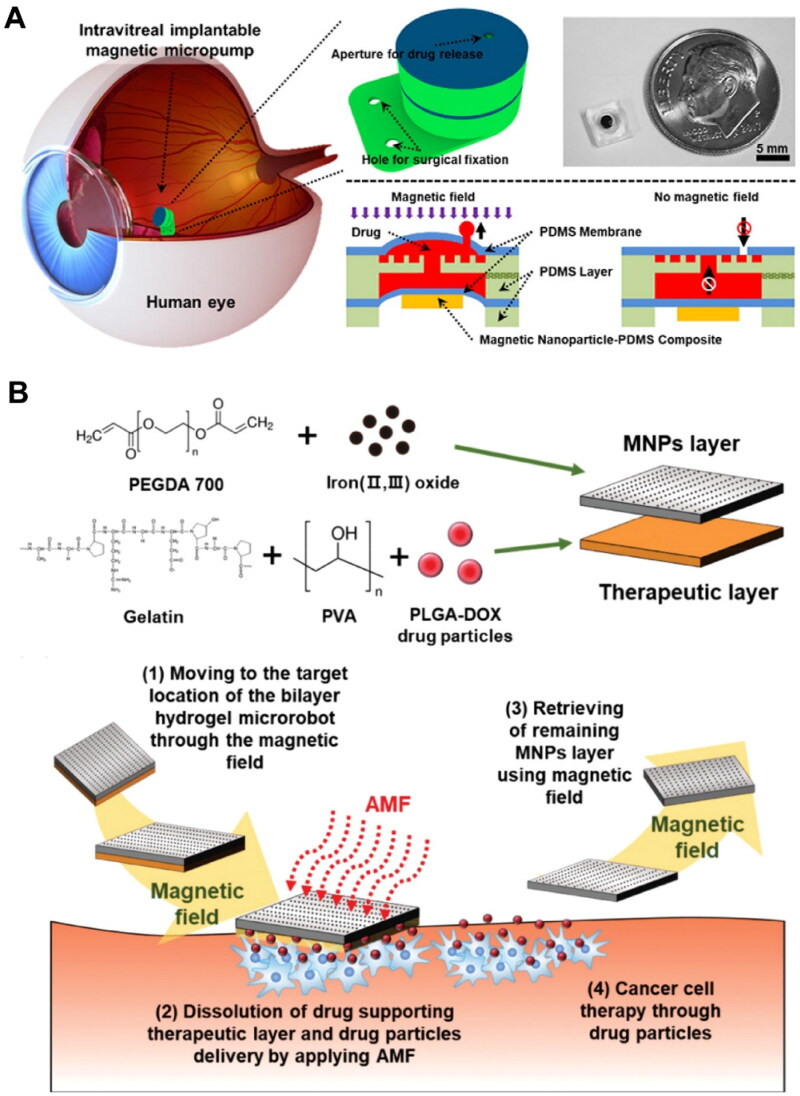
Representative examples of magnetic field-responsive drug delivery for the treatment of posterior segment eye diseases. (A) The working mechanism and drug release profile of an intravitreal implantable magnetic micropump for controlled release of vascular endothelial growth factor receptor (VEGFR) inhibitor. The open check valve enables the upward release of the encapsulated anti-Flt1 gold nanoparticles under a magnetic field, while the closed check valve prevents the payload diffusion. PDMS, polydimethylsiloxane. Reproduced from Wang *et al*. (2018) with permission. Copyright 2018 Elsevier B.V. (B) Schematic illustration of the composition of an intravitreal bilayer hydrogel microrobot and its treatment process. DOX, doxorubicin; AMF, alternating magnetic fields. Reproduced from Kim *et al*. (2020) with permission. Copyright 2020 WILEY-VCH verlag GmbH & Co. KGaA.

Magnetic membrane assembly has been widely employed in the fabrication of magnetically responsive intravitreal implants (Karimi *et al*. 2016). For example, magnetic membranes can serve as the check valves for drug reservoirs (Wang *et al*. 2018). In 2014, Humayun *et al*. first demonstrated the feasibility and safety of the micropump drug delivery system for intravitreal drug delivery in patients with diabetic macular edema during a 90-day follow-up (Humayun *et al*. 2014). Seven patients achieved therapeutic endpoints for maintaining visual acuity following the surgical implantation of the replenished micropump implant. However, the remaining four patients still required additional intravitreal administration as complementary treatment (Humayun *et al*. 2014). Wang *et al*. further utilized magnetic devices to encapsulate anti-Flt1 gold nanocomplexes, enabling wireless control over the release of anti-VEGFR drugs to the macular regions of rabbits (Figure 5A) (Wang *et al*. 2018). However, the implantation and retrieval of such devices usually require invasive surgical procedures (Kar *et al*. 2022). As a step forward, Wang *et al*. reported contact lenses carrying magnetic micropumps (Wang and Park 2020). Upon applying an external magnetic field, the check valve of the embedded micropumps opened and allowed for controlled unidirectional drug release. These smart contact lenses, with noninvasive characteristics, have the potential to improve patient compliance (Kar *et al*. 2022, Wang and Park 2020).

Recently, magnetic nanoparticles (MNPs) and microrobots have been increasingly explored for spatially localized drug delivery through magnetic field guidance (Karimi *et al*. 2016, Veiseh *et al*. 2010). Significant progress in magnetic navigation systems now enables the wireless manipulation of magnetic implants with sub-millimeter precision (Vergne *et al*. 2022). The most commonly used MNPs are iron oxide nanoparticles, which are biocompatible, easy to fabricate, and have been approved as contrast agents for magnetic resonance imaging (Dadfar *et al*. 2019). Magnetically guided intraocular delivery of hydrophobic drugs and genes to the posterior segment using MNPs has been shown to be highly efficient in *ex vivo* porcine eyes and rabbit models (Noh *et al*. 2024). Notably, 6 h of exposure to a permanent magnetic field (around 1.2 T) significantly enhanced the gene transfection in retinal layers after intravitreal injection of adeno-associated viruses (AAVs) attached to MNPs in *ex vivo* porcine eyes. As a comparison, AAVs alone exhibited poor transfection without MNPs due to the hindrance by the viscous vitreous humor and the tight ILM layer (Ahn *et al*. 2024). Noh *et al*. reported that effective delivery of dexamethasone-loaded silica-coated MNPs to the retina could be achieved by placing a magnet on the rabbits’ heads (around 300 mT of magnetic intensity) (Noh *et al*. 2024). In a recent study by Wang *et al*., a hybrid biomembrane-coated iron oxide nanorobot exhibited an 8.1-fold increase in retinal accumulation at 75 min post-intravitreal injection in *ex vivo* porcine eyes when exposed to a 175 mT magnetic field. This nanorobot was used to improve the vitreous transport of nitric oxide-generating agents for treating retinal vein occlusion in rats (Wang *et al*. 2025). However, most studies still relied on intraocular administration, particularly for gene delivery. Bassetto *et al*. found that magnetic fields significantly enhanced the messenger RNA (mRNA) transfection in the retina following subretinal injection of mRNA-encapsulated MNPs, but topical application of these MNPs failed to achieve desirable results (Bassetto *et al*. 2023).

Additionally, the magnetocaloric properties of magnetic materials have been harnessed to achieve precise control over drug release when combined with thermosensitive materials (Chiu-Lam and Rinaldi 2016). Recently, Kim and colleagues developed a microrobot hydrogel implant composed of iron oxide nanoparticles and a therapeutic layer of doxorubicin-encapsulated polymeric nanoparticles (Figure 5B) (Kim *et al*. 2020). By applying an alternating magnetic field, heat generated by the iron oxide nanoparticles caused the dissolution of the supporting hydrogel layer, facilitating the vitreous migration of drug-carrying microrobots. Afterward, the residual iron oxide nanoparticles could be retrieved by permanent magnetic field guidance. This design leveraged the magnetic locomotion of iron oxide nanoparticles to accurately deliver the therapeutic drug to retinoblastoma lesions while addressing the issue of the residual inorganic materials in the vitreous (Kim *et al*. 2020). Additionally, hyperthermia induced by MNPs may provide additional therapeutic benefits for treating ocular tumors, such as retinoblastoma (Demirci *et al*. 2019).

Further biosafety studies are warranted for the use of inorganic materials (Raju *et al*. 2011). Considerable efforts have been made to improve the biocompatibility of magnetic nanoparticles, including surface coating with biocompatible polymers and functionalization by targeting moieties (Cardoso *et al*. 2018). It is widely recognized that the pharmacokinetic profiles and interactions of MNPs with ocular tissues depend on their physicochemical properties (Lyu *et al*. 2021). According to prior research, polystyrene-coated iron oxide microparticles larger than 15 μm could obstruct the trabecular meshwork, along with minor but consistent toxicity on the corneal endothelium for up to 5 months (Raju *et al*. 2011, Schneider-Futschik and Reyes-Ortega 2021). These phenomena were not observed with 50 nm nanoparticles (Raju *et al*. 2011).

### 3.4. Electric field-responsive drug delivery

Electric field-responsive drug delivery systems are promising candidates for ocular drug delivery, as drug transport can be effectively manipulated with simple electrical circuit instruments (Huang *et al*. 2018). These systems can be fabricated using a wide range of electro-conductive or electro-erodible materials, including conductive polymers (e.g., polypyrrole, polythiophene, polyaniline, and silicone), conductive metal materials (e.g., gold, copper, zinc oxide, and ferrocene), carbon nanomaterials (e.g., carbon nanotubes and graphene), and metal-organic frameworks (MOFs) (Sun *et al*. 2020, Zhao *et al*. 2016). However, the development of electric field-responsive drug delivery systems for ophthalmic applications is still in its early stages. Recent research has focused on iontophoresis, which enhances drug transport across biological barriers using low-intensity electrical current flow (Sun *et al*. 2020, Guy *et al*. 2000). This method has long been used to enhance drug penetration and control drug delivery rates in transdermal drug delivery (Pikal 2001). A growing number of studies have utilized iontophoresis to improve the topical or transscleral delivery of small molecules, macromolecules, and nanoparticles to targeted ocular sites (Huang *et al*. 2018, Guy *et al*. 2000, Moisseiev *et al*. 2016, Bejjani *et al*. 2007, Myles *et al*. 2005, Eljarrat-Binstock and Domb 2006). In addition, the electroporation technique has been tested in experimental settings to improve the transfection yield of non-viral genes, including plasmids, RNA, and antisense oligonucleotides (Huang *et al*. 2018, Bejjani *et al*. 2007). This method utilizes transient and high-intensity electric fields to induce reversible permeation of biological barriers, thereby facilitating gene transfer (Bejjani *et al*. 2007).

#### 3.4.1. Iontophoresis-aided drug delivery

The driving mechanisms of iontophoretic transport rely on the physicochemical properties of ionic species and electric field conditions (Huang *et al*. 2018, Guy *et al*. 2000). For small-molecule agents and highly charged polyelectrolytes, iontophoresis-aided drug delivery primarily relies on electro-repulsion, meaning that ionic compounds can be attracted to the opposite electrode (Sun *et al*. 2020, Guy *et al*. 2000). For neutral compounds and macromolecules with a low charge-to-mass ratio, electroosmosis and passive diffusion are the primary contributing mechanisms behind mobility (Guy *et al*. 2000). In these cases, a convective flow of solvent is generated to facilitate their movement when a voltage difference is applied across charged biological membranes (Guy *et al*. 2000). Traditional iontophoretic devices use metallic materials, such as gold, silver and platinum, for electrode fabrication (Huang *et al*. 2018, Seo *et al*. 2023). Carbon, conductive polymers (e.g., poly(3,4-ethylenedioxythiophene) and polypyrrole), and composite of different materials have been utilized for electrode fabrication owing to their improved corrosion resistance and electrical conductivity (Huang *et al*. 2018, Zhang *et al*. 2024).

Topical ionic circuit devices (e.g., eye cups and drug-saturated hydrogels) have been developed to enhance transcorneal delivery to intraocular tissues, particularly for macromolecules and charged nanoparticles with intrinsically poor tissue penetration (Perez *et al*. 2020, Zhang *et al*. 2017). In an early investigation of charged nanoparticles for intraocular delivery via hydrogel iontophoresis, researchers found that negatively charged nanoparticles could rapidly penetrate the cornea in 30 min after iontophoresis treatment (1.5 mA, 5 min), and then slowly migrated into the posterior segment (ciliary body, choroid and retina) in 12 h (Wei *et al*. 2023). Iontophoresis has also been applied for transcorneal gene delivery (Bejjani *et al*. 2007). Asahara *et al*. reported that phosphorothioate oligodeoxynucleotides could move into the vitreous cavity within 5 min and enter the retinal layers within 10 min with the aid of iontophoretic application (1.5 mA) (Andrieu-Soler *et al*. 2006). In addition, iontophoresis at a low direct current (10 μA, 20 min) resulted in a 6-fold increase in transfection efficiency after intravitreal delivery of AAV vectors in mouse eyes (Song *et al*. 2019). The transfection efficiency of intravitreal AAV was also significantly improved in rabbit and nonhuman primate models using iontophoresis at 800–850 μA for 20 min (Song *et al*. 2020).

Notably, transscleral iontophoresis is more commonly applied to improve the transport of suprachoroidally administered agents to the retina, showing great potential for improving drug delivery in the treatment of posterior segment eye disorders (Moisseiev *et al*. 2016, Bejjani *et al*. 2007, Myles *et al*. 2005). Custom-manufactured iontophoretic transscleral applicators involve drug-saturated hydrogels, including OcuPhor and Visulex (Eljarrat-Binstock and Domb 2006). Molokhia *et al*. demonstrated that electroosmosis played a pivotal role in iontophoresis-aided delivery of macromolecules with a relatively low charge-to-mass ratio to the posterior pole of the eye (Molokhia *et al*. 2020). In their studies, iontophoretic transport of immunoglobulin G (IgG) resulted in a 600-fold increase in the delivered amount through the conjunctiva and sclera at a relatively low ionic strength (around 1.8 mA/cm^2^) compared to passive diffusion (Molokhia *et al*. 2020). The iontophoretic delivery of bevacizumab effectively suppressed CNV for 4 weeks after a single treatment in a rabbit model (12.5 mg/mL, 1.4 mA/cm^2^, 20 min), without apparent damage except for mild and short-term inflammation (Molokhia *et al*. 2020). Besides, Jung *et al.* reported that iontophoresis-aided delivery (0.14 mA, 3 min) facilitated the penetration of polystyrene nanoparticles into the innermost suprachoroidal space (30%), achieving about 2-fold higher delivery percentage than that without iontophoresis (Jung *et al*. 2018).

**Figure 6.**
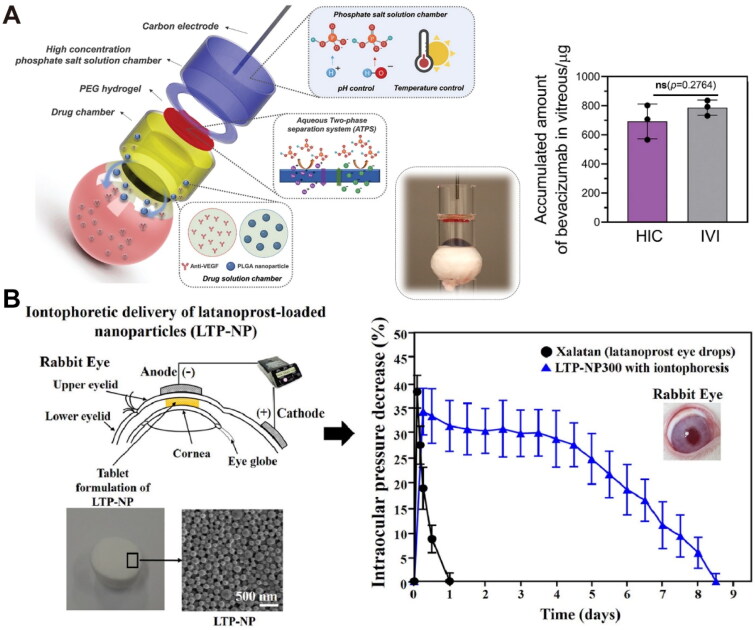
Representative examples of electric field-responsive drug delivery for the treatment of posterior segment eye diseases. (A) Schematic illustration of a hydrogel ionic circuit (HIC)-based iontophoresis device for high-intensity transscleral iontophoresis and quantitative analysis of the accumulated bevacizumab in ocular tissues after high-intensity transscleral iontophoresis (100 mA, 20 min) (*n* = 3). Reproduced from Zhao et al. (2022) with permission. Copyright 2021 Wiley-VCH GmbH. (B) Schematic representation of an iontophoretic device for transcorneal delivery of latanoprost-loaded nanoparticles and profiles of intraocular pressure of rabbit eyes after the treatments with various latanoprost formulations with or without iontophoresis (*n* = 4). Data were presented as mean ± standard deviation. Reproduced from Kim et al. (2022) with permission. Copyright 2022 Elsevier B.V.

Conventional iontophoresis has a low safe current threshold (7.5 mA/cm^2^), due to the potential risks of pH changes from electrolysis and heat generated by electrochemical reactions at high current intensities (Zhao *et al*. 2022). To improve the safety of ocular iontophoresis and enhance its efficiency in delivering macromolecules, Zhao *et al*. constructed a novel hydrogel ionic circuit (HIC) utilizing high-concentration phosphate salt solutions with high conductivity (Figure 6A). The HIC-based iontophoretic device demonstrated improved safe current intensities (up to 100 mA, 87 mA/cm^2^) with minimal Joule-heating side effects (Zhao *et al*. 2022). It enabled the diffusion of therapeutic concentrations of dexamethasone and bevacizumab from the conjunctiva to the vitreous during iontophoresis treatment (100 mA, 20 min), achieving levels comparable to direct intravitreal injection (Zhao *et al*. 2022). To avoid the side effects of direct current stimulation, Qin *et al.* fabricated a wearable electro-driven switch (WES) to enhance protein penetration through the sclera-choroid-retina pathway. WES achieved comparable protein delivery efficiency to intravitreal injection in rabbit eyes using pulse current stimulation (0.73 Hz, 80 µA), which temporarily disrupted the outer BRB and generated electrophoresis effects (Qin *et al*. 2025).

Combining sustained-release nanocarriers with the iontophoresis technique presents a promising approach to maintain therapeutic concentrations after iontophoretic delivery and reduce the number of treatments (Kim *et al*. 2022, Chopra *et al*. 2012, Souza *et al*. 2015). Kim *et al*. prepared a rapidly dissolving polyvinyl alcohol tablet for iontophoretic delivery of latanoprost-loaded polymeric nanoparticles with particle sizes ranging from 100 to 500 nm (Figure 6B) (Kim *et al*. 2022). During noninvasive iontophoresis (4 mA, 30 min), increasing the particle size led to more sustained release of latanoprost, but it also resulted in poorer tissue penetration of the nanoparticles (Kim *et al*. 2022). The 300-nm nanoparticles outperformed other nanoparticles with the most sustained drug concentrations in aqueous humor for over 7 days (Kim *et al*. 2022). Chopra *et al*. proposed using micelles as effective nanocarriers for the iontophoretic delivery of hydrophobic drugs, enhancing their aqueous solubility and enabling prolonged release (Chopra *et al*. 2012). After transscleral transport (2 mA, 20 min), sodium taurocholate-egg lecithin mixed micelles achieved about 55% dexamethasone release over two days (Chopra *et al*. 2012).

#### 3.4.2. Electroporation-aided drug delivery

Electroporation, a technique that uses high-intensity (> 100 V/cm) yet transient (< millisecond) electric fields to induce reversible permeabilization of biological membranes, has been utilized alone or in combination with iontophoresis to improve ocular gene therapies (Huang *et al*. 2018, Bejjani *et al*. 2007). Compared to direct injection of plasmids, electroporation (8 pulses, 200 V/m, 10 ms) can achieve up to 1000 times more gene expression across the cornea (Blair-Parks *et al*. 2002). Electrically assisted gene delivery may offer an efficient and long-lasting therapeutic strategy, particularly for fundus neovascularization and inherited retinal diseases (Bejjani *et al*. 2007, Brar *et al*. 2024). Previous research has demonstrated efficient and sustained gene expression in rats for over 112 days after a single subretinal injection of ­plasmids combined with electroporation-aided transfection (Kachi *et al*. 2006). Touchard *et al*. reported that a notable reduction in laser-induced CNV could be observed within 15 days after a single suprachoroidal injection of a plasmid encoding a soluble vascular endothelial growth factor receptor-1 (sFlt-1) followed by electroporation (8 pulses, 40 V/cm, 20 ms) (Touchard *et al*. 2012). The reporter gene expression peaked on day 7 and, more importantly, could still be detected after 4.5 months (Touchard *et al*. 2012). Aside from gene transfer, efforts have also been made to enhance the ocular delivery of macromolecules and cell vesicles using electroporation (Deora *et al*. 2007, Mandal *et al*. 2018, Hwang *et al*. 2023). Hwang *et al*. investigated the effects of electroporation on improving the retinal delivery of mesenchymal stem cell-derived exosomes after a bolus intravitreal injection (5 pulses, 40 V/cm, 50 ms) (Hwang *et al*. 2023). The results indicated improved uptake of exosomes by the retina, highlighting the potential of this method for treating retinal degeneration (Hwang *et al*. 2023).

So far, some early clinical trials of transscleral iontophoresis-assisted corticosteroid delivery have demonstrated general safety and tolerability for treating inflammation and pain after cataract surgery and noninfectious scleritis (Perez *et al*. 2020, Gratieri *et al*. 2017). At present, there are few clinical investigations of transcorneal iontophoresis, probably due to its low cost-effectiveness compared to other topical formulations (Wei *et al*. 2023). Nonetheless, given the extreme sensitivity of the eyeball, biosafety issues remain regarding the application of electric fields, including temporary damage to ocular tissues and the risks of overheating effects (Zhang *et al*. 2017). More long-term biosafety assessments of these electrically triggered drug delivery systems are still needed before they enter clinical stages.

## 4. Summary and future perspectives

Due to the complex physiology of the eye, delivering drugs to the posterior ocular lesions remains challenging. External stimuli, including light, ultrasound, magnetic and electric fields, have been employed to achieve spatial and temporal control over nanocarriers’ targeting profiles and drug release rates. Furthermore, ultrasound, electric field, and magnetic field have been utilized to improve tissue penetration of nanocarriers and enable drug administration through noninvasive or minimally invasive methods, such as topical applications and intravenous injections. Additionally, various stimuli-responsive nanomaterials with inherent functionality can provide extra clinical benefits, thereby enhancing therapeutic efficacy and reliability in treating complex eye disorders.

Despite the great potential of external stimuli-responsive drug delivery strategies, challenges remain ahead for their clinical applications. Firstly, more efforts must be devoted to understanding the *in vivo* kinetics and final clearance routes of nanomaterials in the body. Systemic absorption and lymphatic clearance are common routes for ocular drug elimination, raising biosafety concerns about the systemic toxicity of nanomaterials (Del Amo *et al*. 2017, Gaudana *et al*. 2010). Additionally, long-term exposure to nanomaterials may cause retinal toxicity and oxidative stress, which requires further evaluation, especially for metallic materials (Zhu *et al*. 2019). Notably, biosafety concerns remain regarding the potential damage caused by external stimuli themselves, including magneto-mechanical interactions and laser burns (Wang *et al*. 2023, Zhang *et al*. 2023). However, most current FDA regulations only provide general guidelines. Given that the eye is a highly sensitive and delicate organ, more thorough biosafety assessments are needed for the therapeutic use of external stimuli on the eye. Although ultrasound- and electrical field-enhanced drug delivery strategies have shown promising results in overcoming ocular barriers, their long-term safety profiles require further investigation, including examination of potential secondary tissue reactions and damage to the BRB integrity.

Secondly, the applications of external stimuli often need sophisticated instrumentation. For example, ionic circuits and ultrasound devices are required to generate electrical fields and ultrasound induction, respectively. These requirements undermine the cost-effectiveness and accessibility of such stimuli-based applications. Light irradiation procedures, on the other hand, are convenient and less painful, making the development of photoresponsive platforms a promising direction for ocular drug delivery. In addition, complex manufacturing processes are required for many proposed drug delivery systems. Ensuring the scalability and reproducibility of these drug delivery platforms is a prerequisite for their clinical translation.

Finally, significant discrepancies in anatomical barriers and drug clearance dynamics often result in gaps between experimental assessments and clinical outcomes for therapeutic evaluations. Rabbits and rodents are most commonly used as experimental models for ocular pharmacokinetic and therapeutic studies (Zeiss 2013, Del Amo and Urtti 2015). Of note, the size of mouse eyeballs is only one-eighth relative to those of human beings, and the mouse vitreous volume (around 5.3 μL) is significantly smaller compared to that of human eyes (around 4.5 mL) (Fan *et al*. 2023). Rabbit eyes also have a lower blinking frequency than human eyes (Del Amo and Urtti 2015, Fan *et al*. 2023, Watsky *et al*. 1988). Many current effectiveness evaluations have only been performed in rodent models. These experimental results may be over-optimistic when translating to large animals and human beings, given the significant species differences.

Collectively, recent achievements in external stimuli-activatable platforms have demonstrated great promise in improving ophthalmic therapeutics, particularly in improving drug delivery efficiency to the posterior segment of the eye. A better understanding of their characteristics, *in vivo* fate, and industrial scale-up potential will contribute to the rational design of smart nanotherapeutics and facilitate their clinical application in the foreseeable future. Cross-disciplinary innovations in ophthalmic medicine, materials science, and biomedical engineering are expected to accelerate groundbreaking progress in ocular nanomedicine, with the aim of to restoring vision in safer, more convenient, and more effective ways.

## Data Availability

Data sharing is not applicable to this article as no new data were created or analyzed in this study.
